# Case Report: Chronic hepatitis E virus Infection in an individual without evidence for immune deficiency

**DOI:** 10.3389/fimmu.2023.1183859

**Published:** 2023-06-19

**Authors:** Dong Ying, Wenxia Niu, Yanling Chen, Yingbin Wang, Weikun Tian, Xiaoping Zhang, Chang Liu, Siling Wang, Zihao Chen, Yajie Lin, Shaoqi Guo, Zihao Yu, Xiuting Chen, Mujin Fang, Hongsheng Qiang, Yifan Yin, Zimin Tang, Zizheng Zheng, Lijuan Fu, Ningshao Xia

**Affiliations:** ^1^ State Key Laboratory of Molecular Vaccinology and Molecular Diagnostics, National Institute of Diagnostics and Vaccine Development in Infectious Diseases, School of Public Health, School of Life Sciences, Xiamen University, Xiamen, China; ^2^ Department of Infectious Disease, Xiang’an Hospital of Xiamen University, Xiamen, China; ^3^ National Medical Products Administration (NMPA) Key Laboratory for Research and Evaluation of Infectious Disease Diagnostic Technology, School of Public Health, Xiamen University, Xiamen, China; ^4^ Xiamen Quality Control Center of Infectious Diseases, Xiamen, China

**Keywords:** hepatitis E virus, chronic hepatitis E, immunocompetent, travel-related, chronic hepatitis B

## Abstract

Chronic hepatitis E virus (HEV) infection occurs mainly in immunosuppressed populations. We describe an investigation of chronic HEV infection of genotype 3a in an individual without evidence for immune deficiency who presented hepatitis with significant HEV viremia and viral shedding. We monitored HEV RNA in plasma and stools, and assessed anti-HEV specific immune responses. The patient was without apparent immunodeficiency based on quantified results of white blood cell, lymphocyte, neutrophilic granulocyte, CD3+ T cell, CD4+ T cell, and CD8+ T cell counts and CD4/CD8 ratio, as well as total serum IgG, IgM, and IgA, which were in the normal range. Despite HEV specific cellular response and strong humoral immunity being observed, viral shedding persisted up to 10^9^ IU/mL. After treatment with ribavirin combined with interferon, the indicators of liver function in the patient returned to normal, accompanied by complete suppression and clearance of HEV. These results indicate that HEV chronicity can also occur in individuals without evidence of immunodeficiency.

## Introduction

Hepatitis E (HE) is caused by the Hepatitis E virus (HEV), which infects 20 million humans worldwide every year, with 3.4 million manifesting symptoms (1). Eight genotypes of HEV have been described to date, and at least five genotypes can infect humans (2). Only zoonotic genotypes are usually associated with chronic infection. HEV causes chronic hepatitis in immunocompromised patients, including patients with solid-organ transplantation, individuals that have undergone stem-cell transplantation, those with hematological disorders, individuals infected with HIV and having low CD4 counts, and patients with rheumatological diseases receiving immunosuppression therapies (3). The majority of chronic cases are asymptomatic, presenting mild to moderate derangement of liver function, and few patients even have normal or near-normal liver enzyme levels. Moreover, the anti-HEV IgM and IgG responses vary among patients with persistent hepatitis E. The absence of seroconversion has been described, despite the presence of viral replication in some persistently infected patients (4), and seroconversion of both IgM and IgG is thought to be more common throughout chronic HEV infection (5–7).

Few cases of chronic HEV infection have been reported in the non-immunosuppressed population, while the immune status of patients has not been totally investigated, and the course of HEV has not been monitored in detail (8, 9). Whether immunocompetent individuals can develop persistent HEV infection and how specific humoral and cellular immune responses in chronic HE patients are to HEV viral pathogens are still unknown. Here, we report a chronic hepatitis E patient who was shown no evidence for immune deficiency and developed persistent HEV infection, despite the presence of a high level of serum anti-HEV IgG with strong neutralization capacity, and these symptoms were resolved after ribavirin combined with PEG-IFN antiviral treatment.

## Case description

**Figure 1:**
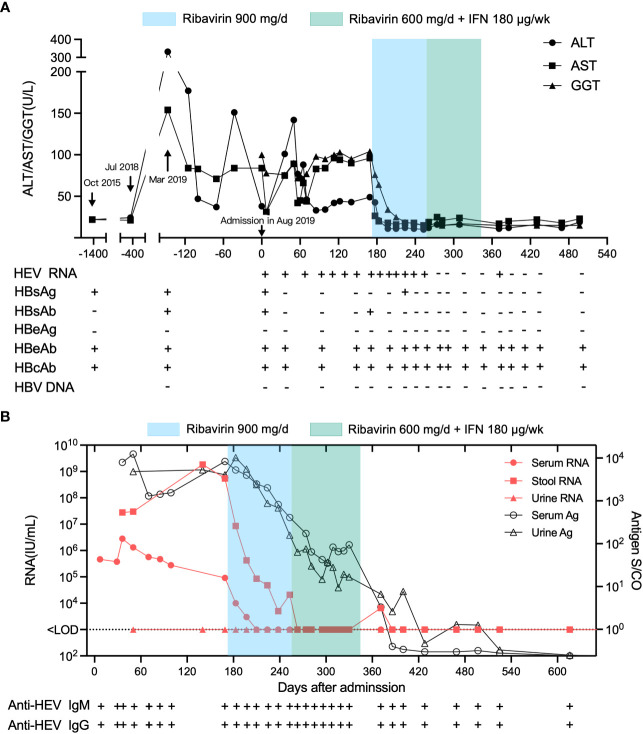
Clinical course of inactive chronic HBV superinfected with HEV in a 63-year-old man at Xiang’an Hospital of Xiamen University before and after treatment. The admission date of the patient in August 2019 was set as day 0. **(A)** ALT, AST, and GGT levels, anti-HEV and anti-HBV serological status, and HEV RNA and antigen status from October 2019 to January 2021. **(B)** HEV RNA concentrations in patient serum and stool and HEV antigen levels in patient serum, stool, and urine over time.

A 63-year-old man was admitted to Xiang’an Hospital of Xiamen University, Xiamen, China, for hepatitis on August 17, 2019. Five months before presentation, his physical examination yielded abnormally elevated transaminase levels (**Figure 1A**); yet, the patient reported no fatigue, anorexia, jaundice, or abdominal pain. Afterward, the patient received oral bicyclol tablets and compound glutathione for hepatoprotective treatment, but his transaminase levels continued to fluctuate above the normal level. The patient visited Xiang’an Hospital of Xiamen University for further diagnosis and treatment at this point.

The patient underwent surgical treatment for tuberculosis of the lumbar transverse process in 1984. After that, he received regular anti-tuberculosis treatment for 1 year and healed well afterward. In 2015, he underwent laparoscopic cholecystectomy due to cholecystitis. Hepatitis B was diagnosed in the same year, with normal transaminase levels, and the patient was diagnosed as being anti-HBe positive at this time (**Figure 1A**, **Table S1**), indicating an inactive phase of chronic hepatitis B (CHB) infection (10). The patient was not treated with antiviral therapy and was followed up regularly. Anti-HBs seroconversion and clearance of serum HBsAg occurred during follow-up. HBV DNA tests were negative when abnormal liver function was found upon physical examination five months before admission(March 2019). The patient had no history of autoimmune disease and denied receiving immunosuppressive drugs. The patient, however, had traveled abroad several times, including the Philippines sixteen months before admission (April 2018), Singapore and Malaysia eight months before admission (November 2018),and Nepal three months before admission (May 2019).

### Biochemical and virological tests

After being admitted to Xiang’an hospital, the patient tested positive for anti-HEV IgM, and both serum and stool samples were positive for HEV RNA. The patient was diagnosed as being in the inactive phase of CHB and being superinfected with hepatitis E. Diagnostic test results for infectious diseases including hepatitis A, C, D and G, HIV, liver fluke, and plasmodium were all negative (**Tables S2**, **S3**). Diagnostic test results for liver-related autoantibodies, including anti-nuclear antibody, anti-mitochondrial antibody, and anti-smooth muscle antibody, were negative, and ceruloplasmin was within the normal range (**Table S2**). His routine blood tests at admission showed a normal white blood cell count (WBC, 8.75 × 10^9^/L), neutrophilic granulocyte count (NEU, 3.23 × 10^9^/L), lymphocyte count (LYM, 3.87 × 10^9^/L), and monocyte count (MON, 0.51 × 10^9^/L) and a slightly increased basophilic granulocyte count (EOS, 1.07 × 10^9^/L; **Tables S2**, **S4**). The serum total IgM, IgG, IgA, and complement (C3 and C4) were all within the normal ranges (**Table S2**). His CD3+ T cell count (1343/µL), CD4+ T cell count (555/µL), CD8+ T cell count (591/µL), and CD4/CD8 ratio (0.94) were also within the normal ranges, indicating that the patient showed no evidence for immune deficiency (**Table S2**).

After the patient was admitted to Xiang’an hospital, his serum, feces, and urine samples were continuously collected for serological testing by ELISA and qRT-PCR (**Figure 1B**). Liver enzymes, including alanine aminotransferase (ALT), aspartate aminotransferases (AST), and glutamyl transpeptidase (GGT), fluctuated more than the normal range after admission, while the patient exhibited normal liver function four years (October 2015) and one year (July 2018) before admission (**Figure 1A**), indicating that his hepatitis was derived from HEV infection. HEV RNA was detected in the serum and feces of the patient at a titer above 10^5^ IU/mL and 10^7^ IU/mL, respectively. The patient was diagnosed with chronic hepatitis E as the patient’s HEV RNA lasted for more than 6 months (**Figure 1B**). Therefore, treatment with 900 mg/d ribavirin was initiated on 170^th^ day after admission (February 4, 2020). Subsequently, 600 mg/d of ribavirin combined with 180 µg/w of PEG-IFN was initiated three months later (April 27,2020). The transaminase and GGT levels returned to normal after 2 and 4 weeks of ribavirin treatment, respectively, indicating the recovery of the patient’s liver function. Serum HEV RNA decreased below the detectable limit after 6 weeks of ribavirin treatment, while fecal HEV shedding persisted for more than 12 weeks after the start of ribavirin therapy. HEV antigen (Ag) levels in the serum and urine decreased after this treatment, consistent with the declining trend of viral RNA.

### Liver histological and immunohistochemical analyses

**Figure 2:**
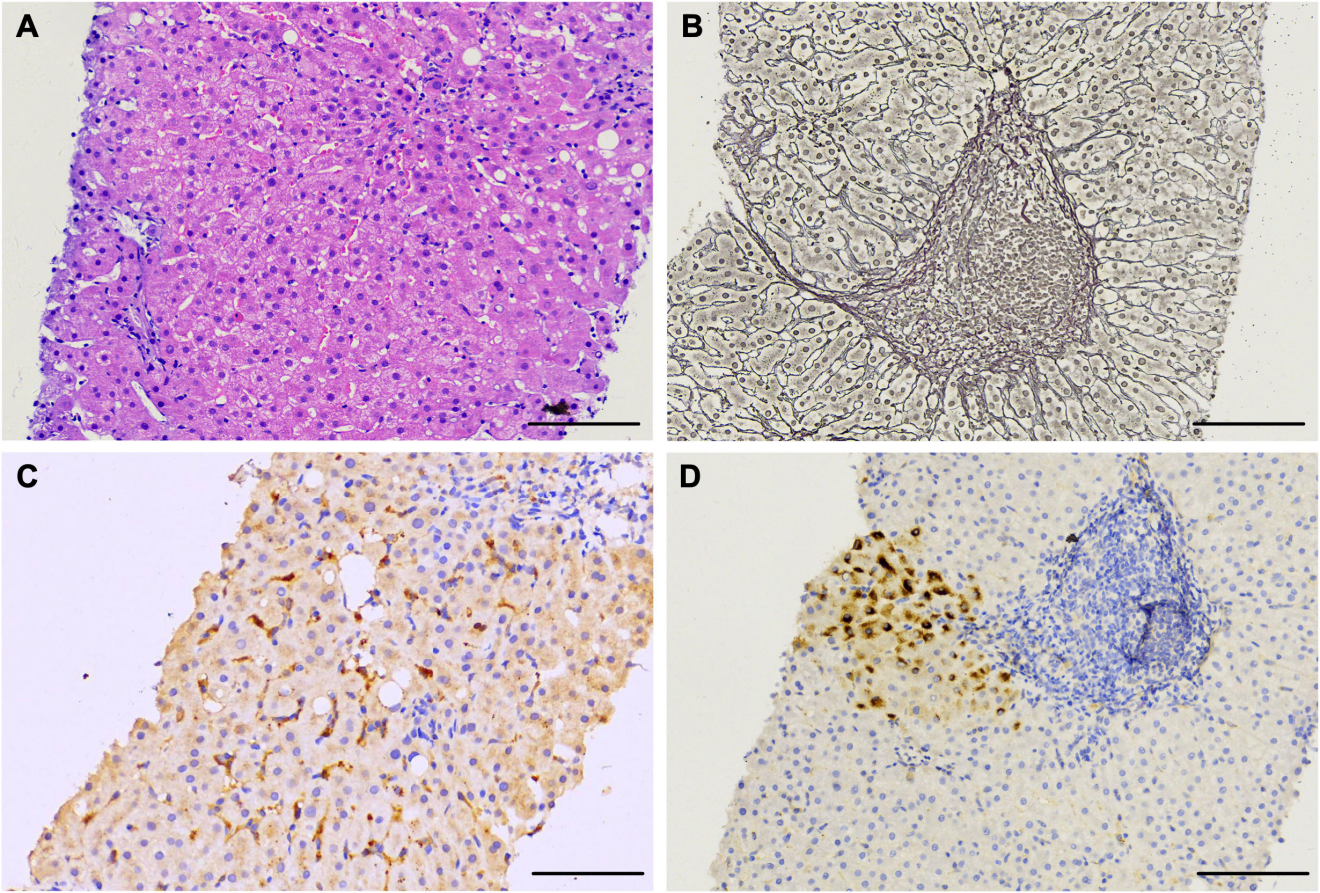
Histological and immunohistochemical staining of live tissue sections of a chronic HE patient obtained at day 56 after admission. **(A)** Liver tissue sections stained with hematoxylin and eosin showing chronic active hepatitis. **(B)** Reticular fiber staining revealing mild hyperplasia of fibrous tissue. **(C, D)** Liver tissue section staining with anti-HEV antibody indicating ORF2 antigen **(C)** and anti-HBV antibody indicating HBsAg **(D)**. Scale bars represent 100 μm.

Liver puncture biopsy was performed two months after being admitted (October 2019), and this revealed the chronic active hepatitis with mild inflammation (METAVIR A1) and moderate fibrosis (METAVIR F1) (**Figure 2**). Regional watery degeneration of liver cells, fatty degeneration of partial liver cells, scattered focal necrosis, and apoptotic bodies were all observed. (**Figure 2A**). An incomplete liver plate in the portal area was observed, with the reticular fibers proliferating and extending to the lobules, suggesting mild hyperplasia of fibrous tissue (**Figure 2B**). Immunohistochemical staining revealed scattered HBsAg positive cells (**Figure 2D**), whereas HBcAg tests were negative (**Figure S1**). Widely distributed HEV Ag was detectable in the liver using a mouse anti-HEV capsid protein antibody (mAb #4, **Figure 2C**), indicating chronic HBV superinfected with HEV.

### Genetic analysis of patient stool samples links infection to Singapore HEV strains

**Figure 3:**
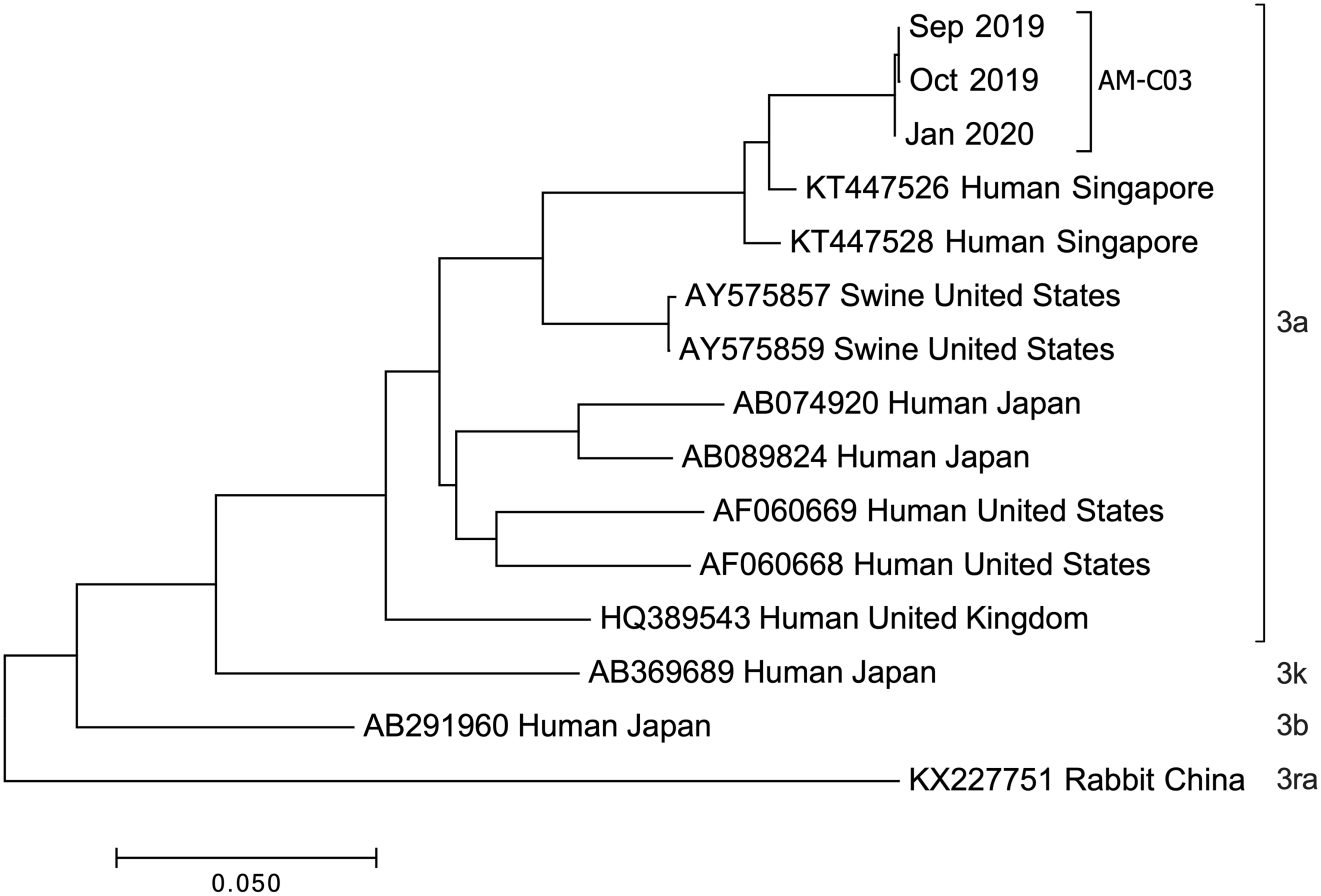
Phylogenetic tree constructed by the neighbor-joining method based on the complete genomic sequence of 12 HEV genotype 3 strains. Genetic distances were calculated using the Kimura two-parameter method. GenBank accession numbers, host, and country of origin are provided. Genome sequences were isolated from chronic HE patient stools at indicated dates.

Complete genome sequencing of HEV isolates from the patient’s stool specimens at several distinct dates was performed, and three genome sequences (Genbank accession OQ801358, OQ801359, OQ801360) were successfully identified. Sequencing showed that the three hepatitis E virus strains belonged to genotype 3a, which was consistent at different sampling time (**Figure 3**). Sequence similarity analysis of the HEV whole genome showed that these isolates were the most identical to the variant KT447526, with 97.0% similarity. Zhu et al. described that the HEV genotype 3a National University Hospital (NUH) clade represented by KT447526 and KT227528 was an endemic local lineage of HEV in Singapore (11). Given the fact that the patient had traveled to Singapore and that his ALT and AST levels were normal a few months before going abroad thirteen months before presentation (July 2018) (**Figure 1A**), we proposed that the patient was infected with HEV in Singapore in eight months before admission into Xiang’an hospital (November 2018).

We also tested the virulence of this HEV strain (**Figure S2**). This strain exhibited stronger infectivity to cells *in vitro* than the virus isolated from an acute patient but had comparable infectivity to a strain isolated from another chronic patient. It is possible that the strain isolated from the chronic patient had replicated in the patient’s body and produced adaptive mutations as we found (**Figure S3**).

### Patient immune status

**Figure 4:**
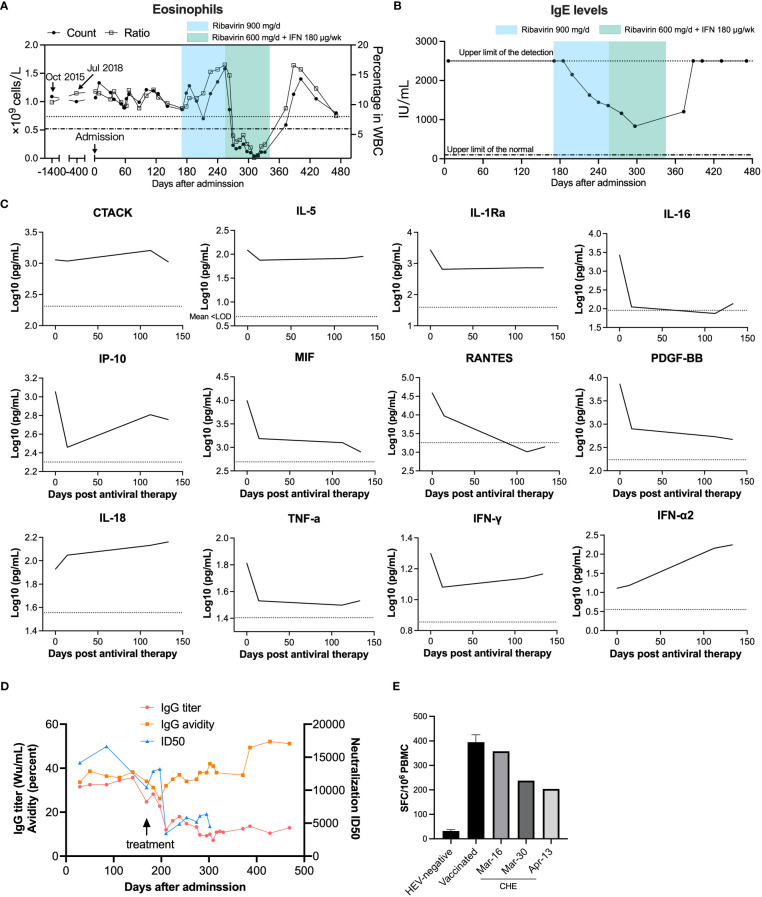
Longitudinal physiological and biochemical tests and cytokine and chemokine profiling of the immune status of an HE patient. **(A)** Eosinophil counts and **(B)** serum total IgE before and after patient first admission. Upper limits of the normal eosinophil count (0.52 × 10^9^/L) and percentage (8.0%) in white blood cell (WBC) are indicated as a dashed dotted line and a dotted line, respectively. The admission date in August 2019 was set as day 0. The normal range of serum total IgE (<100 IU/mL) is indicated with a dashed dotted line. **(C)** Cytokine and chemokine levels before and after antiviral therapy were detected by Bio-Plex assays. The antiviral therapy began on February 4 2020, and this date was set as day 0. Cytokines and chemokines plotted as log10 concentrations over time. Mean concentration of healthy controls is indicated with a dotted line. **(D)** Anti-HEV IgG titers, IgG avidity, and neutralizing capacity before and after ribavirin and interferon treatment. Neutralization of patient serum was determined by the 50% inhibitory dose (ID50). **(E)** Anti-HEV specific T-cell responses were detected by IFN-γ ELISPOT. PBMCs were isolated from this anti-HEV IgG-negative subject and the chronic HE patient and were cultured with HEV 15-mer peptides. IFN-γ secreting cell numbers were counted after stimulation with peptides.

We analyzed the levels of 48 cytokines and chemokines in the patient’s plasma. The patient showed type-2 (anti-helminths) immune responses, with an elevation of the type-2 antibody isotype IgE and the hallmark type-2 cytokine IL-5, which is produced by Th2 cells and ILC2s and is the most potent activator of eosinophils (**Figures 4**, **S4**). To test whether the patient was infected with helminths, we performed eukaryotic 18S ribosome amplicon and metagenome sequencing with blood samples and intestinal samples, but no parasitic pathogen was found (data not shown). Biomarkers for atopic dermatitis, including CTACK (strong correlation), IL-18 (strong correlation), and IgE (moderate correlation), were also increased (12), although the patient had no obvious clinical manifestations of atopic dermatitis. Moreover, the patient showed high serum total IgE at admission. Sustained higher eosinophil levels in the blood of the patient were observed as early as 2015 (**Figure 4A**); then, elevated eosinophils were confirmed by bone marrow examination four months after presentation (December, 2019) (**Table S5**).

The patient showed decreasing serum anti-HEV IgG titer over time from over 30 WHO unit per mL (Wu/mL) before treatment to around 10 Wu/mL, along with reducing viral shedding, after four months of antiviral therapy (**Figure 4D**). The patient also exhibited a notably higher anti-HEV IgG level compared to individuals who were vaccinated with Hecolin^®^ and other acute HE patients and a medium range of IgG avidity (**Figure S5**) (13). The patient showed remarkably high neutralizing capacity with 50% inhibitory dose (ID50) values up to 16,634, in comparison to acute HE patients (**Figure S5C**). The patient’s serum neutralizing activity also decreased over time, which was consistent with the patient’s anti-HEV IgG level. In order to detect which epitopes antibody recognized were induced after chronic infection of hepatitis E virus, we employed competitive ELISA using a panel of murine mAbs that targeting diverse antigenic clusters on the capsid protein as previously described (14). The patient serum HEV specific antibodies predominantly recognize 8G12-like epitope (**Figure S5D**). When compared to acute hepatitis E patients, antibodies recognizing 8C11-like epitope and 8E1-epitope were relatively less dominant in chronic patient serum. These results are consistent with the predominant type of epitopes recognized by antibodies in sera from acutely infected individuals. To test for the generation of anti-HEV specific T-cell responses following infection, samples of the patient’s PBMCs were collected. We stimulated PBMCs from the patient and two anti-HEV IgG negative controls with peptides derived from ORF1, ORF2, and ORF3 of HEV (**Table S6**). HEV specific T-cell responses were detected in the patient’s PBMCs, and these presented a slightly decreasing tendency but were significantly higher compared to HEV-negative controls (**Figure 4E**), indicating a mild to moderate T cell responses to HEV in the chronic HE patient. The T cell response was consistent with the patient’s HEV RNA titer and IgG levels. The overview of the case’s diagnosis, treatment, and follow-up process and timelines of major clinical events are summarized in **Figure S6**.

## Discussion

Since the first reported chronic hepatitis E cases of solid-organ transplant patients by Kamar et al. in 2008 (15), persistent infection caused by HEV has been increasingly recognized and reported in immunocompromised patients. Here, we present a travel-related HEV genotype 3 infected chronic hepatitis E case without immunodeficiency or immunosuppression. Grewal et al. reported a chronic HE case in an immunocompetent patient, but the patient had received immunosuppressive treatment for lupus, and the data were not sufficient to support the immune status of this patient (8, 16). A case of chronic HEV infection in a cirrhotic patient with no apparent immunodepression was reported by Barrague et al., but the patient presented lymphopenia and the immune status before ribavirin treatment was unable to assess (17).Although Wang et al. reported an immunocompetent patient who developed chronic hepatitis E presenting positive for anti-HEV IgM and IgG for 1.5 years, the duration of viremia or viral shedding was not described (9). Our study presented a single strain infected case of chronic hepatitis E with detailed monitoring of viral shedding and anti-viral immune responses from the diagnosis of HEV infection to the complete recovery of the individual. The immune status of the patient was comprehensively determined by the high level of anti-HEV IgG antibodies and positive HEV specific T-cell responses. Absence of HIV, hematological malignancies, and immunosuppressive therapy was conclusively demonstrated in this work. Based on these results, we propose that this was an individual without evidence for immune deficiency who developed chronic HEV infection.

Kemming J et al. reported that chronic HEV infection was associated with specific CD8+ T-cell depletion in immunosuppressed patients (18), suggesting that viral persistent infection may escape T-cell recognition by mutations targeting T-cell epitopes. To explore the T cell epitope mutation in the patient with chronic hepatitis E infection, we found that the HLA-A*11 (in this case) corresponding T cell epitopes did not have any mutation in the amino acid sequence. In addition, it is also possible that the virus strain was more virulent in this patient’s context. Importantly, the presence of a high level of antigen in this patient’s serum, which mainly consists of ORF2^S^ that possesses antigenic epitopes for the capsid protein and inhibits antibody-mediated neutralization, contributes to the escape of the virus from the neutralizing antibodies (19). Several years of chronic infection of HBV may cause the exhaustion of the immune response, which may contribute to the chronicity of HEV infection. Th1 response is necessary for the viral clearance, while the patient showed a Th2 biased response with elevated eosinophilia and IgE, which may be a cause of chronic HEV infection in the patient. Besides, relatively low CD4+ T cell count (555/µL) of the patient may also have elevated susceptibility to HEV infection.

Anti-HEV IgG and IgM antibodies have been detected in some chronic HE patients, while the absence of IgG and/or IgM has also been observed in some patients (4, 7). However, comprehensive analysis of antibody titers, avidity, and neutralizing capacity has not been reported. It is notable that although the patient in our study had high levels of anti-HEV IgG of up to 37.8 Wu/mL with avidity of around 40%, persistently high viral shedding (up to 10^9^ IU/mL) in stool samples was observed before ribavirin treatment. Surprisingly, we found that this patient’s sera had strong neutralizing capacity, raising the question of whether antibodies have limited roles in viral clearance in chronic HE patients. This phenomenon was also observed in a kidney transplanted chronic hepatitis E patient with equivalent IgG titer of up to 35 Wu/mL (20). However, the IgG avidity, serum neutralizing capability, and HEV specific T-cell responses of this patient were not assessed.

There have been too few studies on HEV specific T-cell immunity in acute infection and, in particular, chronic infection. Robust T-cell responses against HEV ORF1, ORF2, and ORF3 proteins were detected in this patient’s PBMCs using IFN-γ ELISPOT assays. These T-cell responses decreased over time, which was consistent with viral shedding in stools and the patient’s antibody titer in serum. Significantly reduced CD3+ and CD4+ T cell counts were observed in HEV persistently infected immunocompromised patients compared to those who had resolved HEV infection (15). T cells may play a role in the clearance of HEV as chronicity of HEV was observed in a moderately immunodeficient patient with decreased lymphocyte counts (21). However, the chronic case in this study presented CD3+, CD4+ and CD8+ T cell numbers that were within the normal range, and an anti-HEV T-cell response comparable with that of the immunocompetent HE patient group was observed, implying new mechanisms for the development of chronic HE.

Our findings have implications for rethinking the development of chronic HEV infection. A major limitation of our study was that we could not define the frequency of chronic infection in immunocompetent individuals. Our previous study and Westholter et al. found that immunocompetent individuals could carry HEV with viremia and antigenemia asymptomatically for 3–4 months, implying that long-term atypical infection of HEV generally exists in the natural population (22, 23). The absence of public health surveillance, however, for asymptomatic HEV infection cases in healthy populations in China and worldwide limits the chance of finding these cases. In conclusion, we have presented the resolved case of a chronic hepatitis E patient treated with ribavirin combined with IFN with detailed kinetics of hepatitis E course and a comprehensive study of humoral and cellular immune responses. We demonstrate that HEV can persist despite the presence of high level of neutralizing antibodies and specific T-cell responses, implying that the potential immune evasion strategies of HEV need further investigation.

## Data availability statement

The datasets presented in this study can be found in online repositories. The names of the repository/repositories and accession number(s) can be found in the article/**Supplementary Material**.

## Ethics statement

The studies involving human participants were reviewed and approved by School of Public Health, Xiamen University. The patients/participants provided their written informed consent to participate in this study.

## Author contributions

DY, ZT, YW, ZZ, and LF designed research. DY, WN, YL,YW, WT, XZ, YC, SG, ZY, XC, MF, HQ, and YY conducted experiments. CL, SW, ZC, and YL contributed analytic tools. DY, ZT and WN analyzed data. DY, YC, ZT, ZZ, and LF wrote the paper. NX supervised the study. All authors contributed to the article and approved the submitted version.
