# Supplementary material for: Case Report: Chronic hepatitis E virus Infection in an individual without evidence for immune deficiency
Source: Front Immunol. 2023 Jun 19;14:1183859. doi: 10.3389/fimmu.2023.1183859 (PMC10315653; doi:10.3389/fimmu.2023.1183859)
Supplement: Supplementary file 1 [file DataSheet_1.docx]

**Supplementary Methods**

*Study design and Patients*

This study was complied with the ethical principles of the Declaration of Helsinki. Ethical approval was obtained from the Ethics Committee of the School of Public Health of Xiamen University. The patient provided written consent to publish this report. Specimens were obtained from the patient at Xiang’an Hospital of Xiamen University after the patient was admitted and diagnosed with HEV infection. Specimens were transported to Xiamen University and stored by refrigeration (2-8 °C) for no longer than 72 h before detection of various indicators. For details on data collection, see the supplementary methods.

*ELISA*

Anti-HEV IgM and anti-HEV IgG were detected using commercial ELISA kits according to the manufacturer’s instructions (Wantai, Beijing, China). HEV antigen was measured with commercial kits provided by Beijing Wantai Biological Pharmacy, which were optimized as previously reported (*1*).

*HEV Complete Genome Sequencing*

RNA was extracted from stool suspension samples using TRIzol and prepared for next-generation sequencing. Briefly, reverse transcription used random hexamers. Subsequent DNase treatment and cleanup were followed by second-strand synthesis before library preparation using Nextera XT reagents (Illumina) and sequencing using a NovaSeq 6000 (Illumina). Although originally described as a consensus-level sequencing methodology, the depth of coverage was such that deep sequencing analysis also could be carried out. Bioinformatics analysis of the data was completed using a pipeline previously described (*2*).

*HEV RNA Detection*

Stool samples were resuspended in PBS at a ratio of 0.2 g/mL w/v. After centrifugation at 12,000 g for 10 min, HEV particles were resuspended in PBS. HEV RNA was extracted from 100 µL of serum samples or stool suspension samples using the Genmag viral DNA/RNA kit and recovered in 50 µL of elution buffer (Genmagbio, Beijing, China). Real-time RT-PCR tests were performed using a CFX96 real-time system and a C1000TM thermal cycler device (Bio-Rad Inc., Hercules, CA USA). JVHEVF:5’ GGTGGTTTCTGGGGTGAC-3’ and JFHEVR: 5’- AGGGGTTGGTTGGATGAA-3’ were used as amplification primers, and JVHEVP: 5’- TGATTCTCAGCCCTTCGC was used as a probe. The RNA value was calculated in International Unit per mL (IU/mL) relative to the standard curve of serial 10-fold dilutions of high-titer plasmids of known potency with viral copy numbers.

*Serum anti-HEV IgG titer, avidity, and neutralizing capacity*

Serum IgG titer was measured against a reference WHO serum (*3*). Anti-HEV IgG avidity was tested as described previously (*4*). Briefly, duplicate serum samples were incubated in Wantai anti-HEV IgG ELISA plates for 30 min at 37°C, with one sample well from the pair following the standard procedure and the other sample well being washed by buffer supplemented with 5M urea, and then, both plates were incubated for 10 min at room temperature. Anti-HEV IgG avidity was the residual antibody level in the presence of urea relative to that in the absence of urea. The initial immune response to viral infection is often characterized by the generation of low-avidity antibodies, which will eventually be replaced by antibodies with higher avidity at the convalescence stage. The serum samples were diluted to have an OD450/620 between 1.0 and 2.0. Serum neutralizing capacity was measured as previously described (*5*). Briefly, 3×10^4^ HepG2/C3A cells were seeded in each well of 96-well plates (PerkinElmer Inc., Waltham, MA, USA) 1 day before inoculation. A mixture of purified nonenveloped Kernow C1/P6 virus (HQ709170) and serial four-fold dilutions (beginning with a dilution of 1:3) of serum samples was incubated for 30 min at 37°C. Mixtures were then inoculated with cells for 6 h at 37°C, followed by three washes with PBS, and the cells were then supplemented with fresh medium containing 10% FBS, 2% DMSO, 100 U/mL penicillin, 0.1 mg/mL gentamicin, and 0.1 mg/mL streptomycin, followed by incubation at 37°C. The cell supernatant was tested for HEV antigen 8 days after inoculation. Neutralization curves were fit using a nonlinear regression model (GraphPad Software Inc., San Diego. CA) to calculate the IC50. The highest concentration will be designated as the IC50 of an mAb or serum if the first dilution displayed less than or equal to 50% neutralizing activity.

*PBMC and T cell response*

PBMCs from blood were isolated with Ficoll-Histopaque by density gradient centrifugation. The cells were cryopreserved in liquid nitrogen until use. HEV-specific T cell responses were measured using a commercial ELISPOT kit according to the manufacturer’s instructions (Cat#: 2110005, Dakewe, Shenzhen, China). Briefly, PBMCs and HEV peptides pool (at a final concentration of 2 µg/mL of each peptide) were added to the plate wells and then were incubated in a 37°C humidified incubator with 5% CO_2_ for 24 h. After the removal of cells, the plate was washed and incubated with detection antibody for 2 h at 37°C. Incubate with streptavidin-HRP for 1 h at room temperature and subsequently develop the plate with substrate solution in the dark for 20 min. Spots were counted in a Series 3B ImmunoSpot analyzer (CTL Analyzers LCC, Cleveland, OH, USA).

*Immunohistochemical and Immunofluorescence Staining*

A liver-punctured biopsy specimen from the patient was fixed through immersion in 4% formalin/PBS for 72h at room temperature. The fixed specimen was embedded in paraffin and sectioned. IHC was performed using an Ultrasensitive^TM^ SP Kit (KIT-9730, Fuzhou Maixin Biotech. Co., Ltd, Fuzhou, China) and a DAB Detection Kit (DAB-0031, Fuzhou Maixin Biotech. Co., Ltd, Fuzhou, China) according to the manufacturer’s instructions. Anti-HEV capsid protein mAb #4 was used as detecting antibody (1mg/mL, 1:1000 dilution). For histopathological analysis, tissue sections were stained with hematoxylin and eosin. All sections were viewed by light microscope BX51 (Olympus, Beijing, China).

*Cytokine and chemokine measurements*

Patient serum was isolated as before and aliquots were stored at −80 °C. Levels of cytokines and chemokines were measured using the Bio-Plex Pro^TM^ Human Cytokine Assays (Bio-Rad Inc., Hercules, CA USA).

*Antibody blocking serum experiments*

D3011 and E09677 were acute hepatitis E patients’ serum samples. 2020/2/18 and 2020/7/14 were two serum samples of the chronic patient. Briefly, selected dilutions of patient serum with an OD value of 1.5 and 20mM phosphate buffer (PBS) were added at 50ul to p239 protein-coated 96-well microplates, followed by incubation at 37 °C for 30 min. Next, unlabeled antibodies recognizing C1-C6 epitopes(6), 20ug per well, were added to the reacted microplate. After incubation at 37 ℃ for 30 min, the microplate was rinsed and a 1:500 dilution of goat anti-human HRP was added. After the last reaction at 37℃ for 30min, the microplate soldiers were rinsed and colored. The blocking rate was measured quantitatively by comparing OD in the presence and absence of competitor mAbs, and transformed using the formula [1- (OD inhibited/OD original)]×100%.

References

1. G. P. Wen *et al.*, A valuable antigen detection method for diagnosis of acute hepatitis E. *Journal of clinical microbiology* **53**, 782-788 (2015).

2. A. Prjibelski, D. Antipov, D. Meleshko, A. Lapidus, A. Korobeynikov, Using SPAdes De Novo Assembler. *Curr Protoc Bioinformatics* **70**, e102 (2020).

3. F. C. Zhu *et al.*, Efficacy and safety of a recombinant hepatitis E vaccine in healthy adults: a large-scale, randomised, double-blind placebo-controlled, phase 3 trial. *Lancet (London, England)* **376**, 895-902 (2010).

4. G. P. Wen *et al.*, Quantitative evaluation of protective antibody response induced by hepatitis E vaccine in humans. *Nat Commun* **11**, 3971 (2020).

5. C. Liu *et al.*, An Optimized High-Throughput Neutralization Assay for Hepatitis E Virus (HEV) Involving Detection of Secreted Porf2. *Viruses* **11**, (2019).

6. M. Zhao *et al.*, A Comprehensive Study of Neutralizing Antigenic Sites on the Hepatitis E Virus (HEV) Capsid by Constructing, Clustering, and Characterizing a Tool Box. *J Biol Chem* **290**, 19910-19922 (2015).

Table S1. Laboratory data of HBV

| Date | HBsAg^a^ | HBsAb^b^ | HBeAg^c^ | HBeAb^d^ | HBcAb^e^ | DNA |
| --- | --- | --- | --- | --- | --- | --- |
| 2015/10/15 | 15.42 | 0.12 | 0.28 | 0.01 | positive | NA^f^ |
| 2019/3/29 | 0.635 | 32.314 | 0.025 | >4 | positive | negative |
| 2019/8/23 | 0.078 | 13.3 | 0.097 | 0.002 | positive | NA |
| 2019/10/8 | 0.024 | 0.004 | 0.029 | 0.028 | 356 | negative |
| 2019/11/12 | 0.021 | 0.006 | 0.029 | 0.030 | 359 | negative |
| 2020/1/6 | 0.022 | 0.008 | 0.048 | 0.040 | 303 | negative |
| 2020/2/4 | ＜0.05 | 28.7 | 0.074 | 0.032 | 293 | negative |
| 2020/3/16 | 0.032 | 0.003 | 0.038 | 0.608 | 98 | negative |
| 2020/3/30 | 0.083 | 5.9 | 0.077 | 0.587 | 101 | negative |
| 2020/4/13 | 0.025 | 0.008 | 0.048 | 0.599 | 87 | negative |
| 2020/4/28 | 0.025 | 0.004 | 0.038 | 0.669 | 94 | negative |
| 2020/5/19 | 0.051 | 0.003 | 0.029 | 0.758 | 74 | negative |
| 2020/5/26 | 0.441 | 0.003 | 0.029 | 0.549 | 80 | negative |
| 2020/6/9 | ＜0.05 | 6.6 | 0.081 | 0.655 | 67 | negative |
| 2020/6/16 | 0.019 | 0.003 | 0.019 | 0.672 | 60 | negative |
| 2020/6/23 | 0.025 | 0.004 | 0.029 | 0.625 | 61 | negative |
| 2020/6/30 | 0.021 | 0.004 | 0.010 | 0.435 | 60 | negative |
| 2020/7/7 | 0.013 | 0.006 | 0.048 | 0.441 | 54 | negative |
| 2020/7/14 | 0.026 | 0.007 | 0.029 | 0.435 | 27 | negative |
| 2020/8/24 | 0.027 | 0.003 | 0.038 | 0.101 | 40 | negative |
| 2020/9/8 | 0.068 | 0.007 | 0.029 | 0.076 | 38 | negative |
| 2020/10/20 | 0.060 | 0.008 | 0.048 | 0.238 | 57 | negative |
| 2020/11/30 | 0.020 | 0.005 | 0.029 | 0.114 | 37 | negative |
| HBsAg^a^, IU/mL, positive: >0.064; HBsAb^b^, IU/L, positive: >13; HBeAg^c^, S/CO, positive: >1; HBeAb^d^, OD value, positive: <2; HBcAb^e^, IU/mL; NA^f^, data not available. | | | | | | |

Table S2. Laboratory test results of this patient upon admission

|  | Reference | Values |
| --- | --- | --- |
| Viral markers |  |  |
| HAV IgM |  | Neg |
| HBeAb |  | Pos |
| HBc IgM |  | Neg |
| HCV Ab |  | Neg |
| HIV Ab |  | Neg |
| HEV RNA |  | Pos |
| HGV Ab |  | Neg |
| Autoimmune |  |  |
| ASMA |  | Neg |
| AMA |  | Neg |
| AMA-M2 |  | Neg |
| LC-1 |  | Neg |
| SLA/LP |  | Neg |
| LKM-1 |  | Neg |
| ANA |  | Neg |
| Parasite |  |  |
| liver fluke |  | Neg |
| blood flukes |  | Neg |
| Immunoglobulin levels |  |  |
| IgA | 0.7 - 4.0 g/L | 0.9 g/L |
| IgE | 0 - 100 IU/mL | >2500 |
| IgG | 7.0 - 16.0 g/L | 15.2 |
| IgM | 0.4 - 2.3 g/L | 1.3 |
| C3 | 0.9 - 1.8 g/L | 0.9 |
| C4 | 0.1 - 0.4 g/L | 0.1 |
| CD3+ T cell | 950 - 2800/μL | 1343 |
| CD4+ T cell | 500 - 1400/μL | 555 |
| CD8+ T cell | 300 - 1200/μL | 591 |
| CD4/CD8 ratio | 0.71 - 2.87 | 0.94 |
| Eosinophils | 0.02 - 0.52 × 10^9^/L | 1.16 × 10^9^ |

Table S3. Laboratory data of renal function

|  | creatinine | urea | cystatin C | eGFR |
| --- | --- | --- | --- | --- |
| reference | 54-106 | 3.2-7.0 | 0.51-1.09 | 90-120 |
| 2019/8/19 | 82 | 4.6 | NA^a^ | NA |
| 2019/10/8 | 82 | 4.8 | NA | NA |
| 2019/10/28 | 92 | 4.9 | 1.19 | 77 |
| 2019/11/12 | 93 | 4.28 | NA | NA |
| 2019/11/26 | 89 | 4.9 | 1.12 | 81 |
| 2019/12/10 | 84 | 6 | 1.18 | 86 |
| 2020/1/6 | 89 | 5.2 | NA | NA |
| 2020/2/4 | 67 | 6.2 | NA | NA |
| 2020/2/12 | 93.18 | 7.73 | NA | NA |
| 2020/2/18 | 87 | 5.8 | NA | NA |
| 2020/3/3 | 89 | 6.6 | NA | NA |
| 2020/3/16 | 97 | 6.5 | NA | NA |
| 2020/3/30 | 100 | 6 | NA | NA |
| 2020/4/13 | 97 | 6.2 | NA | NA |
| 2020/4/28 | 100 | 6.1 | NA | NA |
| 2020/5/6 | 104 | 6.6 | 1.2 | 66 |
| 2020/5/19 | 99 | 5.3 | 1.15 | 70 |
| 2020/5/26 | 85 | 5.6 | NA | NA |

NA^a^, data not available.

Table S4. Laboratory data of blood routine test

|  | WBC^a^ | neutrophils^b^ | neutrophil ratioo | lymphocytes^c^ | monocytes | eosnophils^d^ | eosnophils ratioo | BASO^e^ | HGB | PLT |
| --- | --- | --- | --- | --- | --- | --- | --- | --- | --- | --- |
| reference | 3.5-9.5 | 1.8-6.3 | 40-75 | 1.1-3.2 | 0.1-0.6 | 0.02-0.52 | 0.4-8.0 | 0-0.06 | 120-160 | 125-350 |
| 2015/10/15 | 10.41 | 3.94 | 37.8 | 4.48 | 0.56 | 1.09 | 10.4 | 0.12 | 147 | 161 |
| 2018/7/6 | 8.6 | 3.9 | 45 | 3 | 0.39 | 1 | 11.9 | 0.08 | 162 | 115 |
| 2019/3/29 | 8.96 | 2.89 | 32.22 | 4.47 | 49.9 | 1 | 11.21 | 0.8 | 144 | 147 |
| 2019/8/19 | 8.75 | 3.23 | 37 | 3.87 | 0.51 | 1.07 | 12.2 | 0.07 | NA^g^ | NA |
| 2019/8/26 | 11.21 | 4.11 | 36.6 | 5.02 | 0.65 | 1.33 | 11.9 | 0.1 | NA | NA |
| 2019/9/17 | 10.63 | 3.67 | 34.5 | 5.11 | 0.61 | 1.16 | 10.9 | 0.08 | NA | NA |
| 2019/9/24 | 8.53 | 2.87 | 33.7 | 4.03 | 0.51 | 1.04 | 12.2 | 0.08 | NA | NA |
| 2019/10/8 | 9.19 | 3.4 | 37 | 4.28 | 0.49 | 0.96 | 10.4 | 0.06 | NA | NA |
| 2019/10/14 | 9.23 | 3.29 | 35.6 | 4.42 | 0.57 | 0.89 | 9.6 | 0.06 | NA | NA |
| 2019/10/18 | 10.73 | 4 | 37.2 | 5.01 | 0.49 | 1.05 | 9.8 | 0.07 | NA | NA |
| 2019/10/23 | 9.08 | 4.03 | 44.4 | 3.17 | 0.68 | 1.13 | 12.4 | 0.07 | NA | NA |
| 2019/11/12 | 10.53 | 4.43 | 42.1 | 4.5 | 0.55 | 0.99 | 9.4 | 0.06 | NA | NA |
| 2019/11/26 | 10.52 | 4.02 | 38.2 | 4.64 | 0.56 | 1.21 | 11.5 | 0.09 | NA | NA |
| 2019/12/10 | 9.5 | 3.42 | 36.1 | 4.27 | 0.55 | 1.19 | 12.5 | 0.07 | NA | NA |
| 2019/12/19 | 10.14 | 3.35 | 33.1 | 5.06 | 0.52 | 1.14 | 11.2 | 0.07 | NA | NA |
| 2020/1/6 | 9.05 | 3.27 | 36.1 | 4.32 | 0.46 | 0.92 | 10.2 | 0.08 | NA | NA |
| 2020/2/4 | 9.19 | 3.35 | 36.4 | 4.41 | 0.49 | 0.86 | 9.4 | 0.08 | NA | NA |
| 2020/2/12 | 11.89 | 3.56 | 30 | 6.53 | 0.54 | 1.15 | 9.7 | 0.11 | NA | NA |
| 2020/2/18 | 11.58 | 4.33 | 37.4 | 5.33 | 0.56 | 1.28 | 11.1 | 0.08 | NA | NA |
| 2020/3/3 | 9.07 | 3.6 | 39.7 | 3.94 | 0.47 | 1.01 | 11.1 | 0.6 | 124 | NA |
| 2020/3/16 | 7.42 | 3.22 | 43.3 | 2.92 | 0.35 | 0.7 | 11.9 | 0.05 | 121 | NA |
| 2020/3/30 | 7.35 | 3.32 | 45.2 | 2.52 | 0.34 | 1.14 | 15.5 | 0.4 | 117 | NA |
| 2020/4/13 | 8.47 | 3.9 | 46.1 | 2.76 | 0.41 | 1.35 | 15.9 | 0.05 | 113 | NA |
| 2020/4/27 | 9.45 | 3.75 | 39.7 | 3.6 | 0.44 | 1.58 | 16.7 | 0.08 | 116 | NA |
| 2020/5/6 | 5.79 | 1.43 | 24.6 | 3.09 | 0.4 | 0.86 | 14.9 | 0.01 | 101 | NA |
| 2020/5/13 | 4.77 | 1.65 | 34.7 | 2.52 | 0.36 | 0.23 | 4.8 | 0.01 | 115 | NA |
| 2020/5/19 | 4.45 | 1.61 | 36.2 | 2.34 | 0.32 | 0.17 | 3.8 | 0.01 | 111 | NA |
| 2020/5/26 | 4.59 | 1.57 | 34.3 | 2.49 | 0.33 | 0.19 | 4.1 | 0.01 | 110 | 108 |
| 2020/6/2 | 5.15 | 1.47 | 28.52 | 3.05 | 0.37 | 0.25 | 4.9 | 0.2 | 98 | 98 |
| 2020/6/9 | 3.51 | 1.38 | 39.3 | 1.73 | 0.28 | 0.12 | 3.4 | 0 | 111 | 88 |
| 2020/6/16 | 3.52 | 1.33 | 37.8 | 1.79 | 0.29 | 0.1 | 2.8 | 0.01 | 104 | 93 |
| 2020/6/23 | 3.48 | 1.38 | 39.7 | 1.8 | 0.25 | 0.04 | 1.1 | 0.01 | 115 | 115 |
| 2020/6/30 | 3.25 | 1.26 | 38.8 | 1.7 | 0.23 | 0.05 | 1.5 | 0.01 | 114 | 82 |
| 2020/7/7 | 3.65 | 1.42 | 38.9 | 1.82 | 0.29 | 0.11 | 3 | 0.01 | 112 | 86 |
| 2020/7/14 | 3.3 | 1.43 | 43.3 | 1.49 | 0.26 | 0.11 | 3.3 | 0.01 | 113 | 92 |
| 2020/8/24 | 6.26 | 2.68 | 42.8 | 2.55 | 0.38 | 0.59 | 9.4 | 0.06 | 142 | 130 |
| 2020/9/8 | 6.8 | 2.5 | 36.7 | 2.8 | 0.29 | 1.13 | 16.6 | 0.08 | 157 | 113 |
| 2020/9/22 | 8.81 | 3.4 | 38.6 | 3.54 | 0.38 | 1.4 | 15.9 | 0.09 | 160 | 158 |
| 2020/10/20 | 8.27 | 3.35 | 40.5 | 3.41 | 0.37 | 1.05 | 12.7 | 0.09 | 164 | 154 |
| 2020/11/30 | 9.89 | 4.84 | 48.9 | 3.69 | 0.49 | 0.8 | 8.1 | 0.07 | 159 | 171 |
| 2020/12/28 | 8.11 | 3.43 | 42.3 | 3.34 | 0.39 | 0.87 | 10.7 | 0.08 | 155 | 170 |
|  |  |  |  |  |  |  |  |  |  |  |

WBC^a^, white blood cells, ×10^9^ /L; neutrophils^b^, ×10^9^/L; lymphocytes^c^, ×10^9^/L; eosnophils^d^, ×109/L; BASO^e^, basophilic granulocyte, ×10^9^/L; HGB^f^, hemoglobin, g/L; NA^g^, data not available.

Table S5. Laboratory data of blood and bone marrow smears

| cell name | bone marrow smear | | blood smear |
| --- | --- | --- | --- |
|  | reference | percentage | percentage |
| primitive blood cell |  |  |  |
| primordial granulocyte | 0.12-0.76 | 1 |  |
| promyelocyte | 0.71-1.93 | 1 |  |
| neutrophilic myelocyte | 5.69-10.71 | 4 |  |
| neutrophilic metamyelocyte | 9.29-16.47 | 5 |  |
| neutrophilic rod-shaped nuclei | 12.39-20.33 | 34 | 1 |
| neutrophilic lobulate nuclei | 10.76-20.10 | 11 | 45 |
| eosinophilic myelocyte | 0.05-0.75 |  |  |
| eosinophilic metamyelocyte | 0.25-1.37 |  |  |
| eosinophilic rod-shaped nuclei | 0.01-0.75 |  |  |
| eosinophilic lobulated nucleus | 0.26-2.36 | 5 | 11 |
| basophilic myelocyte | 0-0.07 |  |  |
| basophilic metamyelocyte | 0-0.08 |  |  |
| basophilic rod-shaped nuclei | 0-0.15 |  |  |
| basophilic lobulated nucleus | 0.01-0.19 | 1 | 1 |
| pronormoblast | 0-0.78 |  |  |
| prorubricyte | 0.24-1.34 |  |  |
| polychromatic normoblast | 5.91-10.81 | 1 |  |
| orthochromatic normoblast | 6.96-14.02 | 8 |  |
| promegaloblastic erythrocytes |  |  |  |
| mesomegaloblastic erythrocytes |  |  |  |
| late megaloblastic erythrocytes |  |  |  |
| protolymphocyte | 0-0.14 |  |  |
| naive lymphocyte | 0-0.36 |  |  |
| mature lymphocyte | 14.39-26.25 | 24 | 38 |
| abnormal lymphocyte |  |  |  |
| protomonocyte | 0.03-0.05 |  |  |
| naive monocyte | 0.05-0.33 |  |  |
| mature monocyte | 0.25-2.03 | 5 | 4 |
| protoplasma cell | 0.02-0.024 |  |  |
| naive plasma cell | 0.06-0.26 |  |  |
| mature plasma cell | 0.22-0.88 |  |  |
| reticular cell | 0.04-0.25 |  |  |
| tissue basophils |  |  |  |
| unclassified cell | 0.06-0.12 |  |  |
| oncocyte |  |  |  |
| megakaryocyte | 7-35 | 0 |  |
| naive megakaryocytes |  |  |  |
| granular megakaryocytes |  |  |  |
| plate megakaryocytes |  |  |  |
| bare nucleus megakaryocytes |  |  |  |

Table S6. Peptides used to stimulate the PBMCs

| Peptides | SEQUENCES |
| --- | --- |
| ORF1 PLP 1 | LVFDESVPCRCRTFL |
| ORF1 PLP 2 | TVELAASPGRLECRT |
| ORF1 RdRp 1 | CRMAAPSQRKAVLST |
| ORF1 RdRp 2 | EELGHRPAPVAAVLP |
| ORF1 MET 0 | FLSRLQTEILINLMQ |
| ORF1 Y&MET | RAVVTYEGDTSAGYN |
| ORF2 149-163 | SGTNLVLYAAPLNPL |
| ORF2 341-355 | LTTTAATRFMKDLHF |
| ORF2 493-507 | YVSDTVTFVNVATGA |
| ORF2 533-547 | SKTFYVLPLRGKLSF |
| ORF3 16-30 | CFCLCCPRHRPASRL |

Figure S1. Immunohistochemical staining of HBcAg of live tissue sections of the chronic HE patient. Scale bar represents 100 µm.


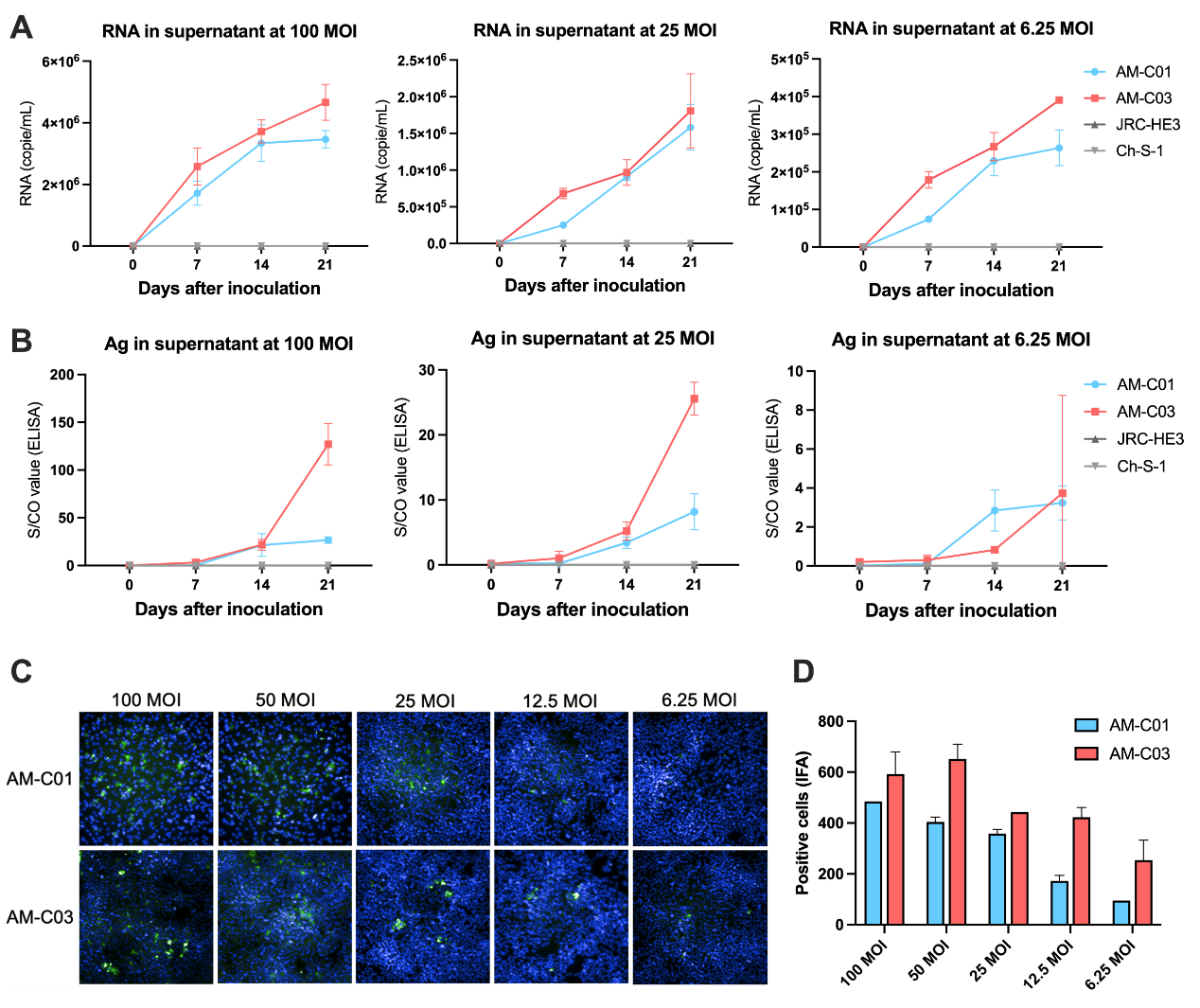


Figure S2. Infectivity of HEV strains isolated from infected patients’ stool in cell culture. (A–B) HepG2 C3A cells were inoculated with a patient stool suspension at indicated MOIs (4-fold serial dilution from 100 to 6.25 genome equivalents per cell). Cell supernatant (A) RNA and (B) Ag were collected and measured every week after inoculation. The viral strain AM-C01 (GenBank: OP185389.1) isolated from an immunocompromised patient and the strain JRC-HE3 (GenBank: AB630971.1) and Ch-S-1(GenBank: EF077630.1) isolated from acute patients were used as a control. Supernatant RNA and Ag were detected by qRT-PCR and ELISA (Wantai, Beijing, China), respectively. Data are shown as the mean ± SEM of triplicate experiments. (C) HepG2 C3A cells were inoculated with virus from these two strains isolated from stools at indicated MOIs (2-fold serial dilution from 100 to 6.25 genome equivalents per cell). Cells were stained with murine anti-HEV antibody 4# (green) and DAPI (blue) at 20 days post-inoculation. (D) Positive cells for ORF2 Ag were counted in each well. Data were gathered from duplicate wells.


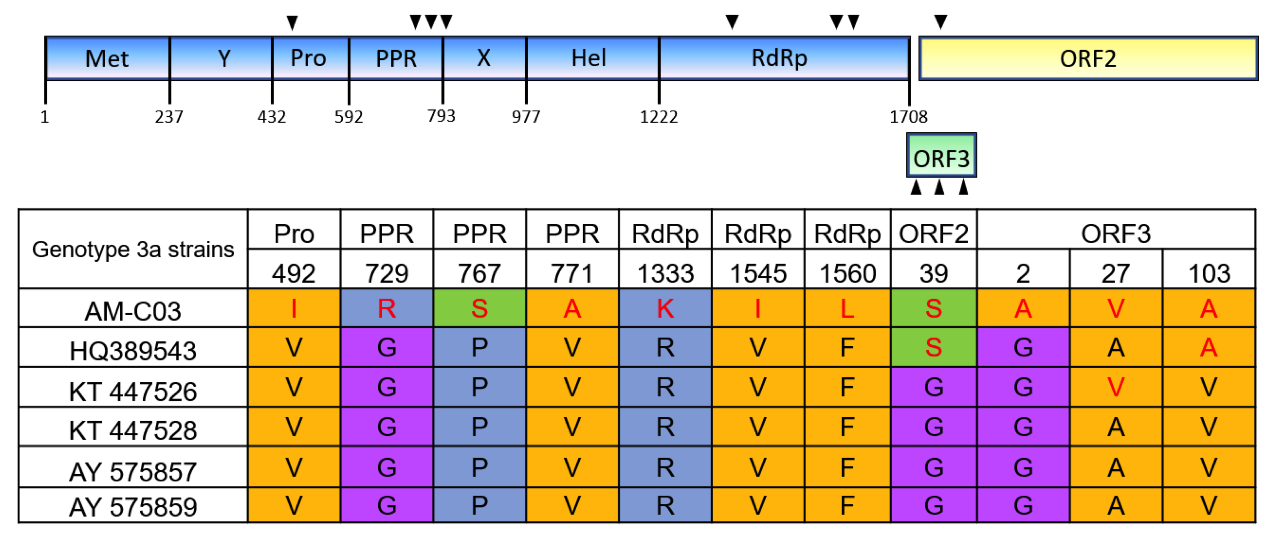


Fig S3. Amino acid sequence analysis of 3 ORFs of the AM-C03 strain. The different amino acid positions of the AM-C03 strain compared with the other genotype 3a strains are shown.

Figure S4. Cytokine and chemokine profiling of the immune status of an HE patient. The value corresponding to the dashed line represents the mean level of cytokine or chemokine of health controls.

Figure S5. The patient’s humoral immune response against HEV. (A-C) IgG titer, IgG avidity and serum neutralization ID 50 of the chronic patient compared to vaccinated serum or acute hepatitis E patients’ serum. (Vaccinated, Hecolin® vaccinated controls; AHE, acute hepatitis E patients; CHE, the chronic hepatitis E patient in this study.) (D) Blocking assay of serum samples to murine monoclonal antibodies. mAb row were blocking parameter of indicated antibodies blocking itself. D3011 and E09677 were acute hepatitis E patients’ serum samples. 2020/2/18 and 2020/7/14 were two serum samples of the chronic patient.


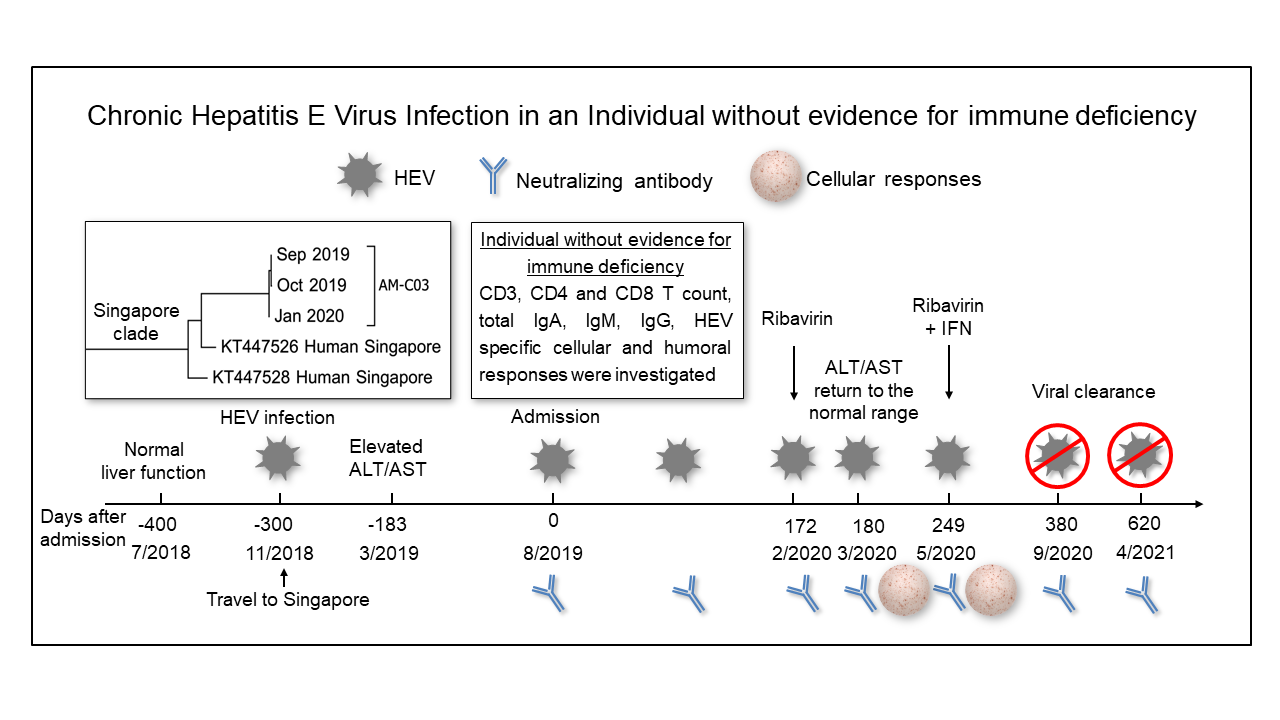


Figure S6. Overview of the case’s diagnosis, management, and follow-up process and timelines of major clinical events.
